# Understanding the Factors that Impact Effective Uptake and Integration of Health Programs in South African Primary Health Care Clinics

**DOI:** 10.21203/rs.3.rs-783631/v1

**Published:** 2021-08-17

**Authors:** Alastair Heerden, Xolani Ntinga, Sheri A. Lippman, Hannah H. Leslie, Wayne T. Steward

**Affiliations:** Human Sciences Research Council Old Bus Depot, KZN; Human Sciences Research Council Old Bus Depot, KZN; University of California San Francisco (UCSF); University of California San Francisco (UCSF); University of California San Francisco (UCSF)

**Keywords:** HIV, primary health care providers, health care facilities, South Africa

## Abstract

**Introduction:**

There is an increasingly urgent gap in knowledge regarding the translation of effective HIV prevention and care programming into scaled clinical policy and practice. Challenges limiting the translation of efficacious programming into national policy include both the paucity of proven efficacious programs that are reasonable for clinics to implement and the difficulty in moving a successful program from research trial to scaled programming. This study aims to bridge the divide between science and practice by exploring health care providers’ views on what is needed to integrate of HIV programming into clinic systems.

**Methods:**

We conducted 20 in-depth interviews with clinic managers and clinic program implementing staff and 5 key informant interviews with district health managers overseeing programming in the uMgungundlovu District of KwaZulu-Natal Province, South Africa. Qualitative data were analyzed using a template approach. A priori themes were used to construct templates of relevance including current care context for HIV and past predictors of successful implementation. Data were coded and analyzed in accordance with these templates.

**Results:**

Heath care providers identified three main factors that impact integration of HIV programming into general clinical care: perceived benefits, resource availability, and clear communication. The perceived benefits of HIV programs hinged on the social validation of the program by early adopters. Wide program availability and improved convenience for providers and patients increased perceived benefit. Limited staffing capacity and a shortage of space were noted as resource constraints. Programs that specifically tackled these constraints through, for example clinic decongestion, were reported as being the most successful. Clear communication with all entities involved in clinic-based programs, some of which include external partners, was noted as central to maximizing program function and provider uptake.

**Conclusions:**

Amid the COVID-19 pandemic, new programs are already being developed for implementation at the primary health care level. A better understanding of the factors which both facilitate and prevent programmatic success will improve public health outcomes. Implementation is likely to be most successful when programs capitalize on endorsements from early adopters, tackle resource constraints, and foster greater communication among partners responsible for implementation.

## Introduction

Antiretroviral therapy (ART) has been established as a highly successful HIV prevention tool, with international calls to prioritize programs that will ensure HIV diagnosis, ART prescription, and viral suppression.(1) Nonetheless, only a small number of programs to improve ART uptake, retention in care, and adherence to medication have been successfully integrated into health systems. Two issues have impeded the successful translation of evidence obtained through research studies into national policy in countries like South Africa with large HIV epidemics. First, relatively few efficacious programs exist that are reasonable to implement in terms of both practicality and cost (2), and second, moving a proven successful program from the context of a randomized trial into scaled programming in public clinics is far from straightforward (3, 4). Health systems often do not have the material or human resources, necessary training, systems set in place, or sufficient buy-in from staff to uptake interventions, particularly in low income, high HIV prevalence countries where clinics are overextended with the growing number of patients initiating and remaining on ART(5–7). Typically these, and other, challenges to broad scale up relate to costs, operational constraints, ensuring quality and consistency of the intervention, and service delivery problems (4). Successful translation of research to practice requires careful consideration and an approach that can facilitate the rapid uptake of relevant skills and incorporation of intervention protocols into the routine delivery of care across the healthcare workforce. Strategies for effective disease prevention and management exist for those who engage in care (8); the challenge is to implement them at scale and in the context of increasingly complex care (9).

There is a growing interest in identifying factors that impact implementation of clinical programming and means to address them at scale, resulting in the development of an array of Implementation Science frameworks (10). Some provide theory to understand aspects of implementation (11), some address research translation to improve quality (12–14), others provide evaluation frameworks to assess implementation success (15), and still others lay out domains of contextual variables to consider as barriers or enablers of implementation, including the Consolidated Framework for Implementation Research (CFIR) (16, 17). These frameworks, largely developed in more resourced settings, are only recently being explored in sub-Saharan Africa (18, 19), where new approaches to ensuring efficient delivery of evidence-based care and innovative interventions across multiple health services are sorely needed.

In low-resource countries where primary care clinics are over extended, there is a clear gap in understanding which contextual factors impact successful program implementation and how these factors can be measured and addressed (20). With over 7.9 million people living with HIV, 71% of whom are engaged in care, South Africa has the largest treatment program in the world (21). These patients require ongoing access to treatment and care; their presence in small primary clinic spaces leads to poor service delivery including long wait times, onerous administrative processes and reduced capacity to adequately care for patients with other conditions (22). These barriers to effective service delivery have been exacerbated by the COVID-19 pandemic and associated social distancing restrictions (23). To address these ongoing challenges, the National Department of Health has introduced several innovative programs over the years which seek primarily to decongest clinics and reduce the amount of time and inconvenience patients experience when picking up their medication. For stable patients, these include fast track initiation, adherence clubs, or formation of peer groups for support and supply of ART outside of the main clinic space, and Centralized Chronic Medicines Dispensing and Distribution (CCMDD), a service allowing for medication pick up in community venues, pharmacies, and other convenient locations to keep clinics ‘decongested’ (24). Programs such as these are based on guidance provided by the South African National Department of Health, influenced heavily by guidelines prepared by the World Health Organization, and are often implemented in collaboration with the President’s Emergency Plan for AIDS Relief (PEPFAR)-funded partner organizations. This qualitative study aimed to understand which factors in the internal and external clinic environment impact effective uptake and integration of health programs in South African primary health care clinics to ensure the programs supporting HIV and other chronic condition are primed for success.

## Methods

### Study Setting

Data collection for this study took place in the uMgungundlovu District of the KwaZulu-Natal Province of South Africa. The district includes the provincial capital Pietermaritzburg and surrounding areas. KwaZulu-Natal has the highest provincial HIV prevalence, with evidence that uMgungundlovu is among the districts with the highest prevalence in the country at 30% (25). The district has 57 permanent health facilities serving just over one million residents with 136,481 residents registered in HIV care (26).

### Study design and Providers

Health care providers and clinic managers were purposively recruited from clinics for In-depth Interviews (IDIs) while program managers and district health officials were invited for Key Informant Interviews (KIIs). Clinics were selected to ensure variability of clinic experiences and patient loads, including both smaller Primary Health Clinics (PHCs) and larger Community Health Centres (CHCs). Within the selected PHCs and CHCs, we recruited up to 2 providers and clinic managers in each for a total of 20 to represent staff currently employed at government primary care clinics where HIV services are delivered. Eligibility criteria for in-depth interviews included being over the age 18; being employed for at least 2 years at a government clinic (either a PHC or a CHC); and being certified to initiate patients who are HIV positive on antiretroviral therapy (ART). We engaged senior staff members, including clinic managers or supervisors, at each clinic, as well as a staff member providing day-to-day care and services at one of the clinics.

For KIIs, sub-district and district HIV management teams were approached to participate. Eligibility criteria included being employed for at least 2 years in a sub-district or district-level management role and being willing to participate. All five key informants had a broad knowledge of program implementation in the local clinics as well as a full understanding of the contextual issues that lead to successful integration of programs targeting people living with HIV (PLWH). They were also selected so as to ensure an intimate working knowledge of the clinic structures and capacity. Approval for the study was obtained from the HSRC Research Ethics Committee and University of California, San Francisco. All participants provided written consent to participate, and the study was approved by the District Department of Health.

### Procedures

IDIs and KIIs followed semi-structured guides and were conducted in Zulu or English by a qualitative interviewer and investigator (XN) fluent in both languages following training and practice interview sessions with the study investigators (WTS, AVH). Following informed consent, the IDIs were conducted at the clinic where the providers were employed and at the offices of the key informants. Interviews were organized to cover two overarching topics. The first aimed to understand the current care context for HIV, including programming available for patients at the clinics who need extra support with retention and adherence, and programming for stable patients. The second topic focused on understanding clinic experiences integrating other national programs for retention in care, including the adherence clubs and clinic decongestion (CCMDD) programs that were integrated into clinic systems beginning in 2017–18. Some clinics failed to integrate these programs and others succeeded. As a result, the interview guide aimed to glean a nuanced understanding of integration successes and failures and what contextual and clinic or provider characteristics played into successes and failures. Topics explored included availability of provider support, supervision, material resources, data systems and training to implement programming as well as “buy in” or political will at both the district and local levels to integrate adherence clubs and CCMDD into integrated care management. All interviews were audio-recorded, transcribed, and translated to English, where necessary, for analysis by the study team.

### Analysis

We analyzed the qualitative interview data using a template analysis approach (27) with templates generated on the following a priori themes of relevance: the current care context for HIV, past predictors of successful implementation of specialized programming, or additional methods of integrating programs into clinic routine. Two investigators (XN, WS) generated an initial coding template within the topical domains relevant to the study. Results were discussed to reach consensus on an initial template with the investigative team. Preliminary coding discrepancies were discussed and resolved, and codes refined as appropriate for a final coding template. When the template was finalized, XN independently coded the data set. Using coded data, the investigators examined convergences and divergences across interviews to thematically identify the key elements for successful integration of programming, as well as challenges and additional considerations.

## Results

### Current care context for HIV

During the interviews, providers identified several specific programs that are considered part of differentiated care delivery for HIV patients. This included CCMDD, adherence clubs, and MomConnect, an mHealth antenatal care program with a component for Prevention of Mother to Child Transmission (PMTCT). Respondents noted that many of the programs are led or supported by collaborating non-governmental organizations (NGOs), including a number of PEPFAR-funded partners with a mandate to implement programs in collaboration with government clinics.

In considering past predictors of program success, providers focused on perceived program impacts as a key factor. Providers described a variety of barriers and facilitators influencing implementation of new programs and services at the clinics. These grouped loosely into those related to the perception of the program and its benefits, resource availability for the program, and communication about organizational roles and functioning of the program. It was also apparent that specific barriers and facilitators were related to one another across the levels of patient, clinic, and system.

### Perceived program impacts

Providers cited the convenience of programs, and specifically the ability of a program to save patient time and money, as being key to that program being well received by patients. For example, fast track queues that reduced clinic wait times for appointments or medications were particularly well received. One provider mentioned that patients love the CCMDD program because of the convenience it gives people to collect their medication without having to miss work or other commitments. This clear benefits to patients was noted as a motivating factor for the providers.

There were limits to the advantages of convenience, however, as reflected by in comments that multiple providers made about adherence clubs. Although the clubs were created to assist with support and medication dispensing, they also had the unintended consequence of patients no longer perceiving a benefit to returning to the facility for any aspect of HIV care. This was a problem because although adherence clubs support routine medication pick-up, they also require bi-annual clinic check-up visits to assess clinical stability and extend the ART prescription. Failing to present for 6-monthly check-ups can result in the unexpected discontinuation of medication until the patient returns to the clinic. One provider also mentioned challenges with adequate club participation. At that clinic, external personnel from a collaborating NGO were traveling from a distant location to assist with the club’s management. Unfortunately, when NGO personnel would arrive at the venue, they would find only a very small number of patients. This occurred despite the clinic and NGO agreeing to open the club on a Saturday to accommodate those with scheduling challenges. Because attendance proved to be so low and the program was inconvenient, the clinic ended up discontinuing the program.

Another factor that impacted provider perception of programs was whether it adequately and appropriately addressed patient needs for privacy. For example, one participant shared similar experiences about the adherence clubs in her facility, saying that the clubs had been the most difficult program for the facility to implement due to the low attendance. A second participant believed that poor attendance at clubs was due in part to stigma, as explained in the following quote:

We do have clubs, but we are lacking in clubs that involve young people like children. We tried to start a group to try and target children because we have a challenge with retaining them to care, but it fell through because of parents, there is still this stigma attached. Parents who bring their children to the clubs get stigmatized that this is a group of parents with HIV positive children.

(Interview: clinic provider 1)

Despite many challenges that hinder program success, providers mentioned that programs that address widely recognized, existing problems, such as overcrowding in the clinic, tend to be more successful. Because the problem is one all providers and staff wish to see solved, there is high motivation to make a potential solution a success, whether it involves implementation of a new program or changes to operational guidelines. For example, one provider described the introduction of universal test and treat (UTT) as a success because clinicians could initiate every eligible patient immediately and thereby simplify the process and reduce risks of opportunistic infections, which had previously been of concern. UTT has also reduced the numbers of people who test but are not linked to care – so is of benefit to the patient and the provider.

I think it was the improvement of the guidelines. Because when you really think about it, we come a long way with the management of HIV patients, before we used to initiate people with CD4 count of 200 and guidelines improved and said we must initiate people with 350, it then went to 500, now we initiate everyone who is tested HIV positive, I think this is a success because we do not have to wait for someone to have a low CD4 count and possibly opportunistic infections…. I also think Universal Test and Treat plays an important role because if you test someone and let them go there is a possibility that they will never come back without being initiated. I think the UTT gives us better result when initiating people.

(Interview: clinic provider 6)

Programs that help decongest the clinic, such as CCMDD, have also been perceived as useful because they foster a more manageable environment that allows for patients whose acuity or need still requires services at the clinic to receive better care. One participant described how clinic decongesting reduces the risk of potential transmission of any airborne illnesses while they wait in long queues.

The advantage is that the patient does not have to sit and wait in long queues, you do not know who you could be sitting next to, the person could have TB or they can be sick you can pick up anything whilst sitting because our facility does not have ventilated areas, so in regard of that even if they are working they are to collect their medication and still go to work, where as if they are in the queue they have got to wait for their place, for blood that is still going to take a little bit longer and they might not even make it to go to work.

(Interview: clinic provider 7)

Providers also mentioned that patient buy-in for a new program was enhanced when patients saw familiar people taking part in it. Seeing someone they recognize helps to facilitate trust and illustrates the potential benefits of a program. One participant mentioned specifically that peer and adult engagement was especially valuable for programs serving young people, as seeing the involvement of others in the community helped foster a safe environment and potential roles models:

It was successful because it helped show the young children that they are not a alone, there’s a lot of them on treatment, they see familiar faces of people they go to school with and so on, and they also see that these people are living a normal life and are alright and don’t have any problems, even younger children who are 9 or 10 years of age who haven’t understood their condition well realize that you can take this treatment and live a normal life, they see the older people here who are taking this and have been for a while.

(Interview: clinic provider 3)

As a result, programs that the providers view as benefitting patients and patient care outcomes and programs that facilitate the providers’ work or workflow were most well received.

### Resource availability

There was wide agreement that shortages of space in clinics poses substantive challenges because of the inability to accommodate all patients who visit the clinic. For example, mothers of children living with HIV all use the same dedicated room on a certain day. This made some mothers uncomfortable due to the gossip associated with walking into that room on the “HIV” day. One participant mentioned that in the clinic where she works, there are patients who hold their adherence club meetings in the medication storage area because there is no other space for them to use.

……they [patients] don’t have places where they can sit and have meetings, and they should have meetings. An adherence club is not a pickup point, people go there to support one another, these people don’t have a place to sit, they have no space available to be utilized for their meetings and they end up going to communities. They also need to get a mobile facility.

(Interview: clinic provider 5)

Staff shortages were also raised as a key resource challenge to successful implementation of HIV programs. Providers described themselves as being overworked by the introduction of new programs, which occurs frequently. The challenges are exacerbated by temporary staffing shortages, such as when staff are on maternity leave. One provider separately noted that staff shortages were also due in part to certain positions being frozen after a resignation.

There will always be challenges whenever there’s something new that’s implemented, it will come with more work for the staff where you find that the staff number is not being increased so the workload increases for the staff of that facility. Sometimes you might see that when we have new things, new guidelines or new programs that are being implemented some of the old ones fall back because we are focusing on this new thing, and we tend to disregard [the old].

(Interview: clinic provider 4)

Providers also mentioned challenges created by paper-based management systems. For patients this can have a very real impact, as a misplaced file can lead to a prescription not arriving at a CCMDD site in time for the patient to refill their medications. Others mentioned that even when and where electronic record technology is available (MomConnect), system failures sometimes compel a return to paper-based record keeping.

### Lines of authority and communication.

There were numerous challenges to clinic implementation of HIV programming that were specific to clear lines of authority, roles and responsibilities, and communication. This poor communication was identified at all levels including patient, provider, and health system. At the patient level, for example, providers had trouble with their abilities to communicate and support patients, which arose in a context of a patient not being comfortable disclosing to family. Clinic personnel felt able to offer only limited assistance because of the precautions required to avoid accidental disclosure when reaching out to patients and/or their family. These challenges were summarized well by one participant:

Some people haven’t even disclosed to their families. So, if the family sends that person to do something on the day where they should pick up their treatment, they cannot say to the family I need to go pick up my treatment. They just go where they are sent and end up missing their pickup. Some have not disclosed even to their partners, you find that someone is visiting their partner and they cannot go to pick up their medication because they do not know what to tell their partner about where they are going.

(Interview: Male district official 1)

Communication issues were not confined to patients, with similar challenges sometimes emerging at the clinic level between provider and NGO staff. Providers agreed that NGO support in the clinics is essential to improving HIV care. Providers noted that NGOs regularly provide counsellors to assist in facilities and NGO staff visit facilities to provide additional clinical and monitoring support. According to the providers, NGOs also aid the clinics by overseeing adherence clubs in communities with high rates of HIV infection and assist with out-of-facility HIV care, including home-based HIV prevention and treatment. One participant described how this help has led to successes with adherence clubs.

We have adherence clubs; it was introduced to us through [name of NGO], they want patients who are HIV positive who are on regimen 1 and are stable patients and the patients need to be able to come collect their own medicine, the purpose of clubs is that patients are able to come collect their medicine and leave quickly without having to sit in the queues for the whole day, which unfortunately does seem to happen. What made it successful is that the guy who’s doing it is pretty dynamic, he’s pretty keen and he pushes hard, he’s got 24 clubs I think or more that he’s managing.

(Interview: clinic provider 8)

Providers also described the role NGOs play in assisting clinics to retain clients in care. NGOs help track people who have missed appointments or have defaulted on treatment and support them to return and remain in care.

I would say it is successful because before we used to lose a lot of patients. But since [name of NGO] started here, they can contact people to ask them why they did not come for their appointments, and they would write down […appointment reminders]. What they do also, they make sure that UTT is implemented, when you test someone, but they do not want to be initiated so they leave their contact details. So, the [name of NGO] team makes sure they contact that person to find out if they are ready to be initiated.

(Interview: clinic provider 9)

While providers generally described the valuable support coming from NGOs, they also noted that there were systems-level challenges in working with these external partners, particularly when there is miscommunication. For example, sometimes NGOs will offer incentives when conducting HIV testing but fail to coordinate with the clinics. As a result, people who are already in care take up this service because they want the incentive, while continuing to return to the clinic for care. Providers also spoke about how the implementing partners responsible for the CCMDD program sometimes create challenges. For example, in some cases only a subset of a client’s total medication needs may be available through an NGO-run CCMDD program. The client is then forced to return to the clinic and request clinic staff to repack and supply them with their medication.

Two providers mentioned communication failures with NGOs related to community testing, noting that NGOs were conducting testing in the communities, but that one was not giving the patients their results.

They [referring to the NGO] would go around testing people from 12 to 25 years old then they would test them and not give them back their results and they would have to come back to the clinic to get their results, bear in mind these were the kids, we didn’t have a clear understanding, we weren’t adequately told about everything and we didn’t know how they were testing these young children, all we knew was that they were taking blood samples, when the young children came to fetch their results it just indicated if they were positive or not in the file, one of them said that they were being tested for STIs so we also ended up confused as to what was happening, and they never came back to inform us on what exactly they were testing.

(Interview: clinic provider 3)

Providers described how the lack of understanding of the local community context can also be a problem when working with NGOs. One participant spoke about an NGO that wanted to create a clinic booking system for patients.

The one thing that they want us to do is have a booking system, whereby you book patients to come and if they don’t come then you phone them then you check why they didn’t come, the problem we have is that patients don’t stick to their bookings, you can tell them to come to that date and they will come a week or 2 later, the problem is that a lot of them work in the informal sector, so they have to take time off, and they don’t know when they going to be off, you can’t plan appointments.

(Interview: clinic provider 8)

## Discussion

Through in-depth interviews we identified that perceived program benefits to patients and workflow, resource availability, and clear communication with health system partners were critical to program implementation successes. While programs that improved convenience for patients and providers, improved health outcomes, and would address known challenges within the facility were taken up more readily, those that had insufficient space and staffing capacity or were more complex or onerous to implement were most likely to fail. Communication and clear roles and responsibilities was important for program success. Informants focused on the promises—and potential challenges—inherent in having clinic partnerships with outside entities. Assistance from local NGOs may help to relieve burdens facing clinic staff, but these same partnerships constitute barriers if communication is vague, and coordination of activities fails to function as intended.

Our research aligns with prior work that has shown that there are substantive variations in the uptake of new programs at clinics in South Africa (29, 30). Other researchers (31, 32) have identified several factors associated with successful or unsuccessful uptake of programs: provider self-efficacy and initiative; skills, resources, and commitment of clinic management; and personal and material resources to integrate programs (33). Our research adds to this literature by showing that the chief implementing facilitator was the perception of clear benefits–both to the patient, in terms of facilitated care, and to the staff, in terms of improved use of time, space, and resources. It suggests that new interventions and practices will be taken up most successfully when there is evident value to all stakeholders. If all stakeholders benefit, the programs will most likely be reported as an unambiguously a win. Other programs with mixed benefits for those involved may be seen either as a total failure or be reported as good in some ways and not so good in others. Indeed, our informants repeatedly highlighted successes with changes such as the rollout of UTT or CCMDD, which have practical and self-evident benefits. For the patients, these practices make available medications known to improve health and save lives (34), while reducing inconveniences to accessing care. For the clinic providers and staff, UTT and CCMDD have simplified treatment protocols, facilitating efforts to retain patients (22) while reducing clinical setting overcrowding, a change that has made it easier to focus on patients with the greatest need. And at the systems level, UTT and CCMDD have given clear roles to different partners, with NGOs leading efforts at HIV testing and medication distribution in community settings while medical providers continue to focus their efforts on clinical environments.

There was more variability in the identified barriers to program implementation. For a clinic’s providers and staff, program uptake is greatly complicated by staffing shortages, which results when people resign or retire and are not replaced. Similarly, a lack of space was recurrently identified as a barrier for new programs and services, as in many facilities there is literally no additional room to place newly created positions or to carve out a location for the delivery of a new service (35, 36). Inadequate resources in turn can undermine the actual or perceived benefits of a program (e.g., limiting times and locations for CCMDD medication pick-up points).

NGOs alleviate some of the resource availability constraints by providing additional human resources, which facilitates additional work either inside or outside a clinic and has been particularly valuable in linking people to care. Despite expressing overall support for the work of the NGOs, our informants repeatedly described instances in which their efforts ended up duplicating or operating at cross-purposes to the work of the clinics due to missunderstandings or poor communication. The finding lends support to the conclusions of Biermann and collegues who argued that an NGO should operate in a manner that is as integrated as possible in existing structure that operate within the community (37). Government clinics report to and receive direction from District, Provincial and ultimately the National Department of Health, while NGOs largely have dual reporting lines both to in coutry Department of Health stakeholders and their funder, typically a US government agencies responsible for local oversight of PEPFAR. Although strategic planning and overall program goals may be harmonised at a national level, the finer details may not always be effectively communicated to all implementers or to local stakeholders, including clinic saff.

Addressing the identified barriers is essential to realize the full potential of HIV care programs. Prior to implementation, programs should be vetted for the characteristics most likely to make them successful, including more in-depth discussions with stakeholders and providers who will ultimately be implementing the program. These discussions will foster local ownership and further increase the likelihood of implementation success. Health officials also need to pay attention to human resource considerations in facilities when planning to implement programs. It is difficult for an understaffed clinic to implement a program that would add more work or supervision responsibilities to personnel who are already struggling to keep up with all their existing responsibilities. For programs to be a success in clinics, they ideally should be addressing challenges recognized as a problem by clinic providers and staff. If a program is only serving the objectives of the external partner, like an NGO, and not addressing a known problem within the clinic, providers and clinic staff may not be intrinsically motivated to go the extra mile to make the program a success. Government and departments of health need to have a more proactive engagement with NGOs working with the clinics to ensure transparency and effective lines of communication to ensure successful program implementation.

### Limitations

There were a number of limitations to this study. First, we had a small sample size, both in terms of total number of providers (25) and number of facilities from which these individuals were sampled (10). This may limit the generalizability of the findings. Second, our methods were restricted to interviews, which rely on providers’ willingness to disclose challenges. We were not able to independently observe clinic operations over time. Third, data were collected once at each facility, raising the possibility that the identified facilitators and barriers are reflecting of temporal trends unique to the current moment but not true of other points in time. Finally, these data were collected prior to the COVID-19 pandemic that began in 2020.

## Conclusions

When time is on the side of the health system, implementation of new programs can often struggle until some level of successes is achieved across the system. Those clinics in which success is achieved often mask the fact that implementation may be incomplete or variable. At this particular point in the history, with the COVID-19 pandemic still an international threat to the health of millions of vulnerable citizens, understanding which factors will aid successful implementation of life-saving programs and policies within the primary health care system need to be urgently distilled and addressed in both ongoing treatment of conditions like HIV and non-communicable diseases and in attending to the threat of new epidemics.

## Data Availability

All data generated or analysed during this study are included in this published article. Any additional data/files may be obtained from the corresponding author.
